# Recent Advances in Image Processing and Computer Vision: Algorithms and Applications

**DOI:** 10.3390/jimaging12080351

**Published:** 2026-08-03

**Authors:** Arslan Munir

**Affiliations:** Department of Electrical Engineering and Computer Science, Florida Atlantic University, Boca Raton, FL 33431, USA; arslanm@fau.edu

## 1. Introduction

Image processing and computer vision continue to play transformative roles across science, engineering, healthcare, transportation, agriculture, manufacturing, environmental monitoring, and intelligent systems [1]. Recent advances in artificial intelligence, machine learning, deep learning, computational imaging, and edge computing have significantly expanded the capabilities of imaging systems, enabling automated interpretation of visual data at unprecedented scales and accuracies [2,3,4,5]. At the same time, fundamental research in image restoration, reconstruction, registration, compression, and representation remains essential for improving image quality and enabling reliable downstream analytics.

This Special Issue, “Image Processing and Computer Vision: Algorithms and Applications”, was launched to provide a forum for researchers to present novel theoretical developments, methodological advances, and practical applications in image processing and computer vision. The contributions span a diverse range of topics, including image enhancement and restoration, medical imaging, remote sensing, human activity analysis, object detection and segmentation, three-dimensional vision, imaging systems, and emerging artificial intelligence techniques for visual understanding. Collectively, these works illustrate the convergence of foundational image processing methodologies with modern artificial intelligence and data-driven computer vision techniques, reflecting the increasingly interdisciplinary nature of contemporary visual computing research.

**Figure 1:**
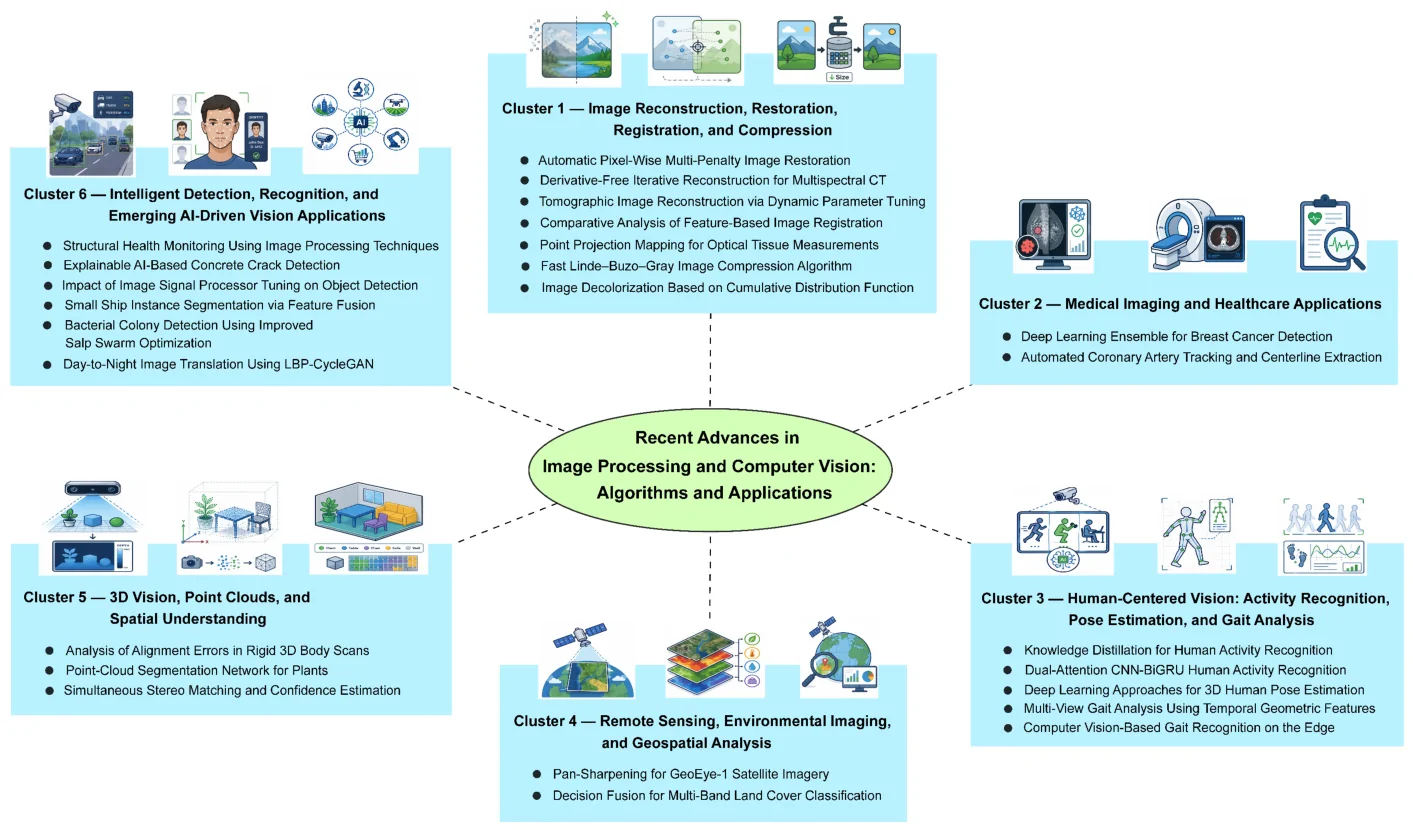
Thematic map of clusters in image processing and computer vision: algorithms and applications.

The papers collected in this reprint volume demonstrate the multidisciplinary nature of contemporary imaging research and illustrate how advances in algorithms, computational models, and intelligent systems continue to drive innovation across numerous application domains. To provide a coherent perspective on this broad and rapidly evolving field, the contributions are organized into six major thematic clusters. Figure 1 provides an overview of this organization, illustrating the structure of the volume across these six interconnected research areas. Collectively, the papers offer a comprehensive view of recent developments in image processing and computer vision, spanning foundational image reconstruction and enhancement techniques, medical and healthcare imaging, human-centered vision, remote sensing, three-dimensional scene understanding, and intelligent AI-driven visual analytics. Beyond highlighting advances within these individual areas, the contributions collectively reveal several cross-cutting research themes, including the growing integration of artificial intelligence (AI) with classical image processing, the increasing emphasis on computational efficiency and real-time deployment, and the expanding role of explainable and trustworthy vision systems across diverse application domains. Together, these studies demonstrate how emerging computational methods are advancing both the theoretical foundations and practical applications of visual computing across diverse scientific and engineering disciplines. The following sections discuss these thematic clusters one by one, synthesize the principal contributions within each area, identify common research trends, and highlight the challenges and future opportunities that emerge across the collected works.

## 2. Image Reconstruction, Restoration, Registration, and Compression

A foundational group of contributions focuses on core image processing methodologies that improve image quality, reconstruction fidelity, computational efficiency, and registration accuracy. Although these studies address different stages of the imaging pipeline, they share a common objective: improving the quality, reliability, and usability of visual information before or during subsequent analysis. Viewed collectively, the contributions illustrate how advances in restoration, inverse-problem reconstruction, spatial alignment, and efficient image representation can provide complementary foundations for downstream computer vision tasks.

The restoration and reconstruction studies approach image quality improvement from distinct but related perspectives. Bortolotti et al. [6] (Contribution 1) introduced an automatic pixel-wise multi-penalty framework for image restoration that removes blur and noise while preserving important image structures. While this approach operates on degraded images through spatially adaptive regularization, Prohaszka et al. (Contribution 2) address image formation more directly by proposing a derivative-free iterative reconstruction method for multispectral computed tomography and tackling the nonlinear inverse problem associated with recovering images from measurement data. Yabuki et al. (Contribution 3) similarly focus on tomographic reconstruction but emphasize dynamic parameter tuning within an iterative optimization framework to improve reconstruction quality under noisy acquisition conditions. Taken together, these studies highlight a broader trend toward adaptive reconstruction and restoration methods that reduce reliance on globally fixed regularization or optimization parameters. At the same time, the different problem formulations demonstrate that no single image-quality enhancement strategy is universally applicable: the appropriate methodology depends strongly on the imaging modality, degradation process, measurement model, and noise characteristics.

The registration-oriented contributions address a complementary challenge: establishing reliable spatial correspondence between images or measurements. Yuan et al. (Contribution 4) presented a comparative analysis of color spaces, detectors, and descriptors for feature-based image registration, providing benchmarking results and practical guidance for registration system design. In contrast to this broad evaluation of algorithmic components, Feenstra et al. (Contribution 5) developed an application-specific point projection mapping system for tracking, registering, labeling, and validating optical tissue measurements in image-guided surgical settings. The relationship between these studies illustrates two important directions in registration research. General benchmarking studies are needed to clarify the performance tradeoffs among alternative feature representations and matching pipelines, whereas domain-specific systems must integrate registration with sensing, tracking, and validation requirements imposed by the target application. Thus, improvements in registration accuracy alone may be insufficient unless robustness and workflow integration are also considered.

The contributions on image compression and representation further emphasize computational and perceptual efficiency. Bilal et al. (Contribution 6) proposed a Fast Linde–Buzo–Gray algorithm that reduces computational complexity while maintaining image quality, reflecting the continuing need for efficient image encoding and storage. Wu et al. (Contribution 7), by contrast, addressed perceptual information preservation during image decolorization, using cumulative distribution functions to better retain contrast and visual details. Although these studies target different operations, both highlight the challenge of reducing or transforming image information without discarding features that are important for human interpretation or subsequent automated analysis. This tradeoff between computational efficiency, compact representation, and information preservation becomes increasingly important as imaging systems generate larger volumes of high-resolution and multimodal data.

Across this cluster, a common challenge is balancing image quality and information preservation against computational complexity, robustness, and application-specific constraints. Reconstruction and restoration methods may require careful adaptation to different acquisition models and noise distributions, while registration pipelines can remain sensitive to changes in appearance, imaging modality, and scene structure. Similarly, compression and image transformation techniques must preserve diagnostically or semantically important information while reducing computational or storage requirements. Future research should therefore investigate more adaptive and modality-aware imaging algorithms, stronger validation under heterogeneous real-world acquisition conditions, and closer integration between low-level image processing and downstream computer vision objectives. In particular, jointly optimizing reconstruction, restoration, or representation methods with subsequent recognition and analysis tasks may help move the field beyond isolated improvements in image quality toward end-to-end imaging systems designed for reliable visual understanding.

## 3. Medical Imaging and Healthcare Applications

Medical imaging represents one of the most consequential application domains for image processing and computer vision, where improvements in visual analysis can directly influence diagnostic support and clinical decision-making. The contributions in this cluster focus on two distinct but complementary medical imaging problems: disease classification from mammographic images and three-dimensional anatomical structure extraction from cardiovascular imaging. At a broader level, these studies also illustrate how the foundational reconstruction, registration, and measurement technologies discussed in the previous section can enable increasingly sophisticated quantitative and learning-based analysis of medical images.

Dehghan Rouzi et al. (Contribution 8) investigated breast cancer detection using an ensemble of deep learning networks with a consensus-adaptive weighting mechanism. Rather than relying on the predictions of a single model, the proposed framework adaptively combines multiple networks to improve mammographic classification performance. Dalvit Carvalho da Silva et al. (Contribution 9), in contrast, addressed coronary artery analysis through a geometry-driven approach and developed an automated Voronoi-based three-dimensional centerline extraction algorithm for coronary artery tracking. Although the two studies differ substantially in methodology—one employing ensemble deep learning for image-level diagnostic classification and the other exploiting geometric structure for anatomical tracking—they share the objective of extracting clinically meaningful information from complex medical imagery with reduced dependence on manual analysis.

The contrast between these contributions is particularly illustrative of the evolving medical imaging landscape. Deep learning offers powerful data-driven mechanisms for learning discriminative patterns that may be difficult to encode explicitly, whereas geometry-based methods can incorporate anatomical structure and domain knowledge directly into the computational pipeline. These approaches should not necessarily be viewed as competing paradigms. Rather, their respective strengths suggest opportunities for hybrid medical imaging systems that combine learned visual representations with anatomical or geometric constraints to improve robustness and clinical interpretability. Moreover, the reconstruction and registration techniques discussed in Section 2 underscore an important dependency within medical imaging pipelines: the reliability of downstream classification, tracking, and quantitative analysis is inherently influenced by the quality and spatial consistency of the underlying image data.

Despite encouraging advances, substantial challenges remain before increasingly automated medical image analysis systems can be broadly translated into clinical practice. Deep learning models are often constrained by limited or heterogeneous annotated datasets, variations across imaging devices and clinical populations, as well as the need for interpretable predictions. Geometry-driven anatomical analysis methods, meanwhile, may be sensitive to image quality, anatomical variability, and pathological structures that deviate from expected geometric patterns. More broadly, rigorous external validation, uncertainty quantification, reproducibility, and integration into existing clinical workflows remain essential considerations. Future research should explore hybrid approaches that combine data-driven learning with anatomical priors, leverage multimodal imaging information, and explicitly account for uncertainty and domain variability. Such advances could help bridge the gap between high algorithmic performance in controlled studies and reliable deployment in real-world healthcare environments.

## 4. Human-Centered Vision: Activity Recognition, Pose Estimation, and Gait Analysis

Understanding human motion, behavior, and activities remains a central challenge in computer vision because visual observations of people are inherently dynamic and affected by variations in viewpoint, appearance, execution speed, and environmental conditions. The contributions in this cluster address human-centered visual understanding at different but interconnected levels, ranging from activity-level interpretation to body-pose modeling and gait-based analysis. Collectively, they reveal two recurring priorities in this area: learning effective spatial–temporal representations of human motion and achieving sufficient computational efficiency and robustness for practical deployment.

The two human activity recognition studies by Ullah and Munir [7,8] (Contributions 10 and 11) illustrate complementary strategies for addressing the tradeoff between representational capability and computational efficiency. Their knowledge-distillation-based framework transfers information from large, three-dimensional convolutional neural networks to lightweight student models, targeting efficient recognition suitable for real-time deployment. In contrast, their cascaded dual-attention convolutional neural network (CNN) and bidirectional gated recurrent unit (GRU) architecture emphasizes richer spatial–temporal feature learning by combining attention-based spatial representation with recurrent temporal modeling. Viewed together, the two approaches highlight a fundamental tension in human activity recognition: increasingly sophisticated architectures can improve the modeling of complex motion patterns, but their computational demands may limit deployment on resource-constrained platforms. Knowledge distillation and other model-compression strategies therefore provide an important bridge between high-capacity activity recognition models and practical real-time systems.

Pose estimation and gait analysis examine human motion through more structured representations of the body. El Kaid and Baïna’s systematic review (Contribution 12) of deep learning approaches for three-dimensional human pose estimation highlights the growing use of learned models to infer articulated body configurations from visual observations. Such pose representations can provide an intermediate abstraction between raw image sequences and higher-level activity interpretation, suggesting a natural connection between pose estimation and human activity recognition. Rather than learning activities exclusively from appearance and motion features, future recognition systems may increasingly exploit explicit skeletal or pose-based representations to improve generalization and interpretability.

The gait-related contributions further demonstrate how temporal and geometric properties of human motion can support recognition and analysis. Pattanapisont et al. (Contribution 13) proposed a multi-view gait analysis framework based on temporal geometric features and majority-voting fusion, directly addressing variations in observation viewpoint. Salcedo (Contribution 14), from a broader systems perspective, surveyed computer-vision-based gait recognition for edge computing environments and emphasized the importance of efficient feature representations, models, and architectures for resource-constrained deployment. These studies are complementary: the former focuses on improving motion representation and robustness across views, whereas the latter highlights the computational and architectural constraints that arise when gait recognition moves from experimental settings to edge platforms. Together with the activity recognition studies, these works demonstrate that model accuracy cannot be considered independently of deployment efficiency and operating conditions.

Several challenges cut across the contributions in this cluster. Human-centered vision systems remain sensitive to occlusion, viewpoint changes, illumination variations, background complexity, and differences in how individuals perform the same activity or gait pattern. Three-dimensional pose estimation also faces depth ambiguity and the difficulty of recovering reliable body configurations from incomplete visual evidence. At the systems level, maintaining recognition accuracy while reducing model size, memory requirements, and inference latency remains essential for real-time and edge applications. Future research should investigate more unified human-motion representations that connect pose, gait, and activity semantics; adaptive models capable of generalizing across viewpoints and environments; and efficient learning frameworks that jointly consider recognition performance and deployment constraints. Privacy-preserving visual analysis and interpretable human-centered models will also become increasingly important as these technologies are adopted in healthcare, biometrics, human–computer interaction, and intelligent environments.

## 5. Remote Sensing, Environmental Imaging, and Geospatial Analysis

Remote sensing and Earth observation continue to benefit significantly from advances in image processing and computer vision, particularly as modern sensing platforms generate heterogeneous data with different spatial, spectral, and temporal characteristics. The contributions in this cluster address a common challenge in geospatial analysis: how to effectively combine complementary information to improve the quality and interpretation of remotely sensed data. Although the two studies operate at different stages of the analysis pipeline, both emphasize the central role of information fusion in extracting more reliable knowledge from multi-source or multi-band observations.

Alcaras and Parente [9] (Contribution 15) investigated the effectiveness of pan-sharpening algorithms across different land-cover categories using GeoEye-1 satellite imagery. By examining algorithm performance as a function of land-cover type, their study demonstrates that the effectiveness of image fusion cannot necessarily be characterized by a single global assessment. Instead, the spatial and spectral properties of the observed scene can influence the suitability of a particular pan-sharpening method. This finding highlights the importance of application- and scene-aware algorithm selection in remote sensing, where heterogeneous landscapes may challenge methods optimized or evaluated under limited environmental conditions.

Papadopoulos et al. (Contribution 16) examined information fusion from a complementary perspective through a comprehensive review of pixel-level decision-fusion approaches for land-cover classification using multi-band remote sensing data. Their analysis encompasses optical, thermal, hyperspectral, and synthetic aperture radar (SAR) imagery and demonstrates how combining decisions derived from complementary sensing modalities can improve land-cover interpretation. While pan-sharpening integrates information primarily to enhance the spatial and spectral representations of imagery, decision fusion combines information at a later stage to improve classification outcomes. Viewed together, the two contributions illustrate how fusion can be incorporated at multiple levels of the remote sensing pipeline, from image formation and enhancement to semantic interpretation and decision-making.

This relationship also exposes an important design question for future geospatial imaging systems: at what stage should heterogeneous information be fused? Early or image-level fusion can provide richer visual representations but may introduce spectral distortion or propagate artifacts into subsequent analysis. Conversely, decision-level fusion can preserve modality-specific processing pipelines but depends on the reliability and calibration of the individual classifiers or decision sources being combined. The contributions in this cluster therefore suggest that the fusion strategy should be selected in consideration of the sensing modalities, land-cover characteristics, and ultimate analytical objective, rather than treated as a universally applicable preprocessing or classification step.

Several challenges remain in developing robust fusion-based remote sensing systems. Differences in spatial resolution, spectral response, sensor geometry, acquisition time, atmospheric conditions, and data quality can complicate the integration of heterogeneous observations. Moreover, algorithms evaluated on particular sensors or geographic regions may not generalize consistently to different landscapes and environmental conditions. Future research should investigate adaptive fusion frameworks that dynamically account for scene characteristics, sensor uncertainty, and downstream task requirements. Closer integration of image-level and decision-level fusion, potentially through end-to-end learning and uncertainty-aware models, may enable geospatial analysis systems that more effectively exploit complementary sensing information while maintaining robustness across diverse environmental and operational settings.

## 6. 3D Vision, Point Clouds, and Spatial Understanding

Three-dimensional (3D) sensing technologies have become increasingly important for industrial inspection, healthcare, robotics, and precision agriculture, where reliable geometric information is essential for measurement, object-level analysis, and spatial decision-making. The contributions in this cluster address distinct but interconnected stages of 3D visual understanding: the quality and alignment of acquired 3D data, the semantic interpretation of point-cloud representations, and the estimation of scene depth, together with confidence in the resulting predictions. Viewed collectively, these studies demonstrate that accurate spatial understanding depends not only on increasingly capable learning algorithms but also on the geometric integrity and reliability of the underlying three-dimensional information.

Meißner et al. (Contribution 17) examined the measurement foundations of 3D analysis by systematically characterizing alignment errors in rigid 3D body scans. Their analysis of translational and rotational misalignments demonstrates how geometric inconsistencies introduced during alignment can affect measurement accuracy. Peng et al. (Contribution 18), in contrast, addressed the semantic interpretation of 3D data through a point-cloud segmentation network for plant phenotyping that combines a SqueezeNet-inspired architecture with temporal information. The transition from the alignment problem studied by Meißner et al. to the segmentation problem investigated by Peng et al. illustrates an important dependency in 3D vision pipelines: errors in geometric acquisition or alignment can propagate into downstream semantic analysis, potentially affecting the reliability of object- or structure-level measurements.

Schmähling et al. (Contribution 19) considered another dimension of reliability by jointly addressing stereo matching and confidence estimation within a multi-task network. While the first two studies focus on the quality and interpretation of explicitly represented three-dimensional geometry, stereo matching requires depth information to be inferred from paired visual observations. Simultaneously estimating disparity and confidence provides a mechanism not only for generating depth information but also for assessing the reliability of those estimates. This emphasis on confidence complements the alignment-error analysis of Meißner et al.; both contributions demonstrate that identifying potential sources of spatial uncertainty is important for interpreting the outputs of 3D vision systems. In this context, the point-cloud segmentation framework of Peng et al. further highlights the downstream importance of reliable geometry, as semantic and instance-level plant analysis ultimately depends on the quality of the spatial representation provided to the learning model.

Together, the three contributions reveal a progression from geometric quality assessment to semantic spatial interpretation and uncertainty-aware depth estimation. They also expose several persistent challenges in 3D vision. Point clouds and reconstructed depth representations can be affected by sensor noise, sparsity, occlusion, alignment errors, and incomplete scene observations, while learning-based models must generalize across variations in object geometry and acquisition conditions. Computational complexity is another important consideration, particularly when dense three-dimensional data must be processed in real time or on resource-constrained platforms. Future research should more closely integrate geometric quality assessment, uncertainty estimation, and semantic analysis so that downstream models can explicitly account for the reliability of their spatial inputs. Adaptive 3D vision systems that propagate confidence or uncertainty information throughout the processing pipeline—from acquisition and alignment to depth estimation and semantic understanding—may provide a promising path toward more robust spatial intelligence in safety- and measurement-critical applications.

## 7. Intelligent Detection, Recognition, and Emerging AI-Driven Vision Applications

The growing integration of AI into computer vision has expanded the range and complexity of visual analysis tasks that can be addressed computationally. The contributions in this cluster span infrastructure assessment, advanced driver-assistance systems, maritime surveillance, biological imaging, and image-to-image translation. Despite their diverse application domains, they share a common theme: the performance and practical utility of intelligent vision systems depend on the interaction among image acquisition and transformation, feature representation, learning or optimization strategies, and the interpretability of the resulting predictions. Collectively, these studies illustrate a shift from viewing the recognition model as an isolated component toward considering the broader visual analytics pipeline in which intelligent inference operates.

The contributions of Kim et al. (Contribution 20) and Swarna et al. (Contribution 21) provide complementary perspectives on intelligent vision for structural health monitoring. Kim et al. reviewed image-processing-based technologies for monitoring civil infrastructure, tracing the increasing integration of imaging systems, machine learning, and AI into inspection and maintenance workflows. Within this broader landscape, Swarna et al. [10] developed a feature-fusion-based framework for concrete crack detection and segregation that incorporates explainable AI to provide interpretable model outputs. The relationship between these studies highlights an important evolution in infrastructure vision systems: increasing detection accuracy alone is insufficient for high-consequence inspection applications, where users may also need to understand the visual evidence underlying automated predictions. Explainability can therefore serve as a bridge between algorithmic performance and the trust required for practical decision support.

Molloy et al. (Contribution 22) and Sharma et al. (Contribution 23) address object-level visual understanding from different stages of the computer vision pipeline. Molloy et al. investigated the influence of image signal processor (ISP) tuning on object detection performance in advanced driver-assistance systems, demonstrating that upstream image processing choices can directly affect the performance of downstream deep learning models. Sharma et al., in contrast, focused on the learning architecture itself by developing an enhanced atrous spatial pyramid pooling feature-fusion framework for small ship instance segmentation. Their approach targets the difficulty of identifying small objects in challenging maritime scenes. Viewed together, these studies demonstrate that detection and segmentation performance is shaped by both the quality and characteristics of the input imagery and the ability of a model to represent objects across challenging spatial scales. This connection suggests that image acquisition, ISP configuration, and model architecture should increasingly be co-designed rather than optimized independently.

The studies by Ihsan et al. (Contribution 24) and Almohamade et al. (Contribution 25) further broaden the methodological landscape beyond conventional supervised detection architectures. Ihsan et al. employed an improved salp swarm optimization algorithm for multi-feature selection in bacterial colony detection and classification, illustrating how intelligent optimization can identify informative visual features for biological image analysis. Almohamade et al. combined local binary pattern (LBP) information with a CycleGAN architecture for day-to-night image translation, emphasizing texture preservation and computational efficiency during visual domain transformation. Although these approaches address fundamentally different tasks, both manipulate the representation of visual information before the final interpretation: one selects discriminative features, while the other transforms image appearance while seeking to preserve important structural and textural content. Their contributions reinforce the broader observation that intelligent visual analysis is strongly influenced by how relevant information is selected, preserved, or transformed prior to downstream inference.

Across this cluster, several challenges emerge from the increasing complexity of AI-driven vision pipelines. Models developed for specific datasets or operating conditions may experience performance degradation under changes in illumination, sensor characteristics, scene composition, object scale, or application domain. Upstream image processing and domain transformation can improve visual quality or normalize appearance, but they may also alter features that are important for downstream recognition. Similarly, increasingly complex feature-fusion and deep learning architectures can improve predictive performance while creating challenges related to computational cost and interpretability. Future research should therefore move toward pipeline-aware and task-aware visual intelligence, in which acquisition, image processing, representation learning, and inference are jointly optimized for the final application objective. Greater emphasis on explainability, uncertainty assessment, cross-domain robustness, and efficient deployment will also be essential for translating AI-driven vision systems from benchmark evaluations to trustworthy operation in infrastructure monitoring, transportation, environmental surveillance, and biological analysis.

## 8. Conclusions and Future Directions

The contributions collected in this reprint volume demonstrate the breadth of contemporary image processing and computer vision research while also revealing important connections across seemingly distinct application domains. From adaptive image restoration and tomographic reconstruction to medical image analysis, human motion understanding, remote sensing, 3D vision, infrastructure monitoring, and generative image transformation, the studies collectively show that reliable visual intelligence depends on the interaction of multiple stages of the imaging pipeline. Image acquisition and reconstruction influence the quality of subsequent analysis; registration and geometric consistency affect quantitative interpretation; feature representation and information fusion shape recognition performance; and computational efficiency, confidence assessment, and explainability increasingly determine whether advanced vision algorithms can be translated into practical systems.

One recurring theme is the movement toward more adaptive and task-aware imaging pipelines. The pixel-wise multi-penalty restoration framework, dynamically tuned tomographic reconstruction, land-cover-dependent evaluation of pan-sharpening methods, and adaptive weighting of deep learning ensembles all challenge the assumption that globally fixed algorithms or parameters are equally suitable across heterogeneous images, sensing conditions, and application scenarios. Similarly, the comparison of feature-based registration components and the investigation of image signal processor tuning demonstrate that upstream processing choices can substantially influence downstream visual analysis. These contributions suggest that future systems should increasingly co-optimize acquisition, reconstruction, enhancement, and inference with respect to the final analytical objective, rather than treating individual processing stages as independent modules.

A second theme concerns the representation and integration of complementary visual information. Spatial–temporal learning in human activity recognition, temporal geometric features for gait analysis, multi-band decision fusion in remote sensing, feature fusion for concrete crack detection and small-ship segmentation, and temporal information in point-cloud analysis all demonstrate the value of combining information across spatial scales, time, sensing modalities, or feature representations. At the same time, the contributions expose an unresolved question: how and at what stage should complementary information be fused? Image-level, feature-level, temporal, and decision-level fusion strategies offer different advantages and limitations. Future research should investigate adaptive and uncertainty-aware fusion mechanisms capable of selecting and weighting information according to scene characteristics, sensor reliability, and task requirements.

Computational efficiency emerges as another cross-cutting requirement, rather than a secondary implementation concern. Knowledge distillation for human activity recognition, edge-oriented gait recognition, the Fast Linde–Buzo–Gray compression algorithm, lightweight point-cloud processing, and computationally efficient day-to-night image translation reflect the growing need to deploy sophisticated visual models under constraints on latency, memory, energy, and processing capacity. However, reducing computational complexity must be balanced against the preservation of the spatial, temporal, geometric, and textural information needed for reliable inference. An important future direction is therefore the development of resource-aware vision systems in which model compression, representation design, and hardware-aware optimization are jointly considered with accuracy and robustness, particularly for real-time and edge applications.

The volume also highlights a broader shift from predictive accuracy alone toward reliability, interpretability, and awareness of uncertainty. The analysis of alignment errors in 3D body scans and the joint estimation of stereo disparity and confidence demonstrate the importance of characterizing the reliability of geometric information. Explainable AI for concrete crack detection similarly illustrates the need to expose the evidence supporting automated predictions in high-consequence inspection applications. In medical imaging, ensemble-based classification and geometry-driven anatomical tracking further emphasize the need for robust analysis under variations in data and anatomical structure. Building on these contributions, future vision systems should more explicitly quantify and propagate uncertainty across the imaging pipeline, provide interpretable outputs appropriate for the target users, and undergo validation under heterogeneous real-world operating conditions rather than relying primarily on performance under controlled benchmark settings.

Finally, the diversity of application domains represented in this volume underscores the increasingly interdisciplinary nature of visual computing. Healthcare, precision agriculture, environmental monitoring, transportation, maritime surveillance, biological analysis, and civil infrastructure impose different sensing conditions, performance requirements, and consequences of error. Yet, the contributions reveal shared methodological challenges involving data quality, domain variability, computational constraints, and trustworthy inference. The next generation of image processing and computer vision systems will likely be shaped by closer integration of adaptive image formation, multimodal and spatial–temporal representation learning, efficient edge intelligence, and uncertainty-aware and explainable AI. Foundation models and emerging generative approaches may further expand these capabilities, but their value in specialized imaging domains will depend on their ability to incorporate domain knowledge, operate with limited or heterogeneous data, and satisfy application-specific requirements for efficiency, reliability, and interpretability.

Taken together, the contributions in this reprint volume suggest that the future of visual computing lies not simply in developing increasingly complex models, but in designing integrated imaging systems in which acquisition, processing, representation, and intelligent inference are considered jointly. We hope that the studies collected here will serve as valuable references for researchers, practitioners, and students, and will stimulate further research toward adaptive, efficient, and trustworthy visual computing systems capable of operating reliably across diverse real-world environments.

## References

[1] Ghazal S., Munir A., Qureshi W.S. (2024). Computer Vision in Smart Agriculture and Precision Farming: Techniques and Applications. Artif. Intell. Agric..

[2] Khan S., Naseer M., Hayat M., Zamir S.W., Khan F.S., Shah M. (2022). Transformers in Vision: A Survey. ACM Comput. Surv..

[3] Minaee S., Boykov Y., Porikli F., Plaza A., Kehtarnavaz N., Terzopoulos D. (2022). Image Segmentation Using Deep Learning: A Survey. IEEE Transactions on Pattern Analysis and Machine Intelligence.

[4] Awais M., Naseer M., Khan S., Anwer R.M., Cholakkal H., Shah M., Yang M.-H., Khan F.S. (2025). Foundation Models Defining a New Era in Vision: A Survey and Outlook. IEEE Transactions on Pattern Analysis and Machine Intelligence.

[5] Trigka M., Dritsas E. (2025). A Comprehensive Survey of Deep Learning Approaches in Image Processing. Sensors.

[6] Bortolotti V., Landi G., Zama F. (2023). An Automatic Pixel-Wise Multi-Penalty Approach to Image Restoration. J. Imaging.

[7] Ullah H., Munir A. (2023). A 3DCNN-Based Knowledge Distillation Framework for Human Activity Recognition. J. Imaging.

[8] Ullah H., Munir A. (2023). Human Activity Recognition Using Cascaded Dual Attention CNN and Bi-Directional GRU Framework. J. Imaging.

[9] Alcaras E., Parente C. (2023). The Effectiveness of Pan-Sharpening Algorithms on Different Land Cover Types in GeoEye-1 Satellite Images. J. Imaging.

[10] Swarna R., Hossain M., Khatun M., Rahman M., Munir A. (2024). Concrete Crack Detection and Segregation: A Feature Fusion, Crack Isolation, and Explainable AI-Based Approach. J. Imaging.

